# Assessment of Physician Priorities in Delivery of Preventive Care

**DOI:** 10.1001/jamanetworkopen.2020.11677

**Published:** 2020-07-27

**Authors:** Jessica J. Zhang, Michael B. Rothberg, Anita D. Misra-Hebert, Niyati M. Gupta, Glen B. Taksler

**Affiliations:** 1 Cleveland Clinic Lerner College of Medicine, Case Western Reserve University, Cleveland, Ohio; 2 Medicine Institute, Cleveland Clinic, Cleveland, Ohio; 3 Department of Quantitative Health Sciences, Cleveland Clinic, Cleveland, Ohio; 4 Center for Health Care Research and Policy, Case Western Reserve University and MetroHealth Medical Center, Cleveland, Ohio

## Abstract

**Importance:**

Primary care physicians have limited time to discuss preventive care, but it is unknown how they prioritize recommended services.

**Objective:**

To understand primary care physicians’ prioritization of preventive services.

**Design, Setting, and Participants:**

This online survey was administered to primary care physicians in a large health care system from March 17 to May 12, 2017. Physicians were asked whether they prioritize preventive services and which factors contribute to their choice (5-point Likert scale). Results were analyzed from July 8, 2017, to September 19, 2019.

**Exposures:**

A 2 × 2 factorial design of 2 hypothetical patients: (1) a 50-year-old white woman with hypertension, type 2 diabetes, hyperlipidemia, obesity, a 30-pack-year history of smoking, and a family history of breast cancer; and (2) a 45-year-old black man with hypertension, hyperlipidemia, obesity, a 30-pack-year history of smoking, and a family history of colorectal cancer. Two visit lengths (40 minutes vs 20 minutes) were given. Each patient was eligible for at least 11 preventive services.

**Main Outcomes and Measures:**

Physicians rated their likelihood of discussing each service during the visit and reported their top 3 priorities for patients 1 and 2. Physician choices were compared with the preventive services most likely to improve life expectancy, using a previously published mathematical model.

**Results:**

Of 241 physicians, 137 responded (57%), of whom 74 (54%) were female and 85 (62%) were younger than 50 years. Physicians agreed they prioritized preventive services (mean score, 4.27 [95% CI, 4.12-4.42] of 5.00), mostly by ability to improve quality (4.56 [95% CI, 4.44-4.68] of 5.00) or length (4.53 [95% CI, 4.40-4.66] of 5.00) of life. Physicians reported more prioritization in the 20- vs 40-minute visit, indicating that they were likely to discuss fewer services during the shorter visit (median, 5 [interquartile range {IQR}, 3-8] vs 11 [IQR, 9-13] preventive services for patient 1, and 4 [IQR, 3-6] vs 9 [IQR, 8-11] for patient 2). Physicians reported similar top 3 priorities for both patients: smoking cessation, hypertension control, and glycemic control for patient 1 and smoking cessation, hypertension control, and colorectal cancer screening for patient 2. Physicians’ top 3 priorities did not usually include diet and exercise or weight loss (ranked in their top 3 recommendations for either patient by only 48 physicians [35%]), although these were among the 3 preventive services most likely to improve life expectancy based on the mathematical model.

**Conclusions and Relevance:**

In this survey study, physicians prioritized preventive services under time constraints, but priorities did not vary across patients. Physicians did not prioritize lifestyle interventions despite large potential benefits. Future research should consider whether physicians and patients would benefit from guidance on preventive care priorities.

## Key Points

**Question:**

How do physicians choose which evidence-based preventive services to discuss with patients?

**Findings:**

In this survey study of 137 primary care physicians, participants agreed that they prioritized preventive services when time constrained (mean score, 4.27 on 5-point Likert scale). Physicians reported prioritizing by potential to improve patient quality or length of life but focused on only 1 of the 3 preventive services most likely to help patients live longer.

**Meaning:**

These findings suggest that physicians may benefit from tools to improve prioritization of evidence-based services.

## Teaser

This survey study assesses primary care physicians’ prioritization of preventive services.

## Introduction

Although professional guidelines encourage preventive care, 32 major evidence-based services are available for nonpregnant adults,^1^ and delivery of all services is difficult.^2,3,4,5,6,7^ Just 8% of US adults attain all guideline-recommended services.^8^ Although patient nonadherence is an important factor, in many practice settings, physicians also have limited time to discuss preventive care.^9,10,11,12^ To fully evaluate and implement all preventive care guidelines, physicians would need to devote an estimated 7 hours per day to primary prevention.^10^ However, clinicians spend just 27% of their day in direct clinical care.^13^ These factors potentially force physicians to prioritize guideline recommendations.

Which preventive services are most often discussed and whether priorities differ by physician remains unknown. Physicians may prioritize care in different ways, such as improving length of life, quality of life, or cost-effectiveness. However, clinical decision supports focused on these priorities are limited. Instead, physicians must rely on personal impressions of the benefits and harms of each preventive service, taking into account differences across patients in risk factors. Mathematical models have been used to rank preventive services based on the potential to add life-years,^14,15^ improve quality of life,^16,17,18^ or lower costs,^16,17,18^ but how these rankings compare with clinical practice is unknown. Finally, prioritization could be more important in short encounters compared with longer annual wellness visits. Our goal was to understand whether primary care physicians prioritize the preventive services they discuss with patients and the factors influencing their priorities, especially visit length, patient risk factors, and potential to improve life expectancy.

## Methods

We used a 27-question anonymous online survey from March 17 to May 12, 2017, and compared physician responses with results of a validated, evidence-based mathematical model of preventive care previously published by one of us (G.B.T.).^14,15^ Analyses were conducted from July 8, 2017, to September 19, 2019. For the survey, we invited all 241 internal and family medicine physicians practicing at the Cleveland Clinic Health System, a large academic medical center with 13 regional hospitals, 21 family health centers, and more than 75 outpatient locations. Physicians were contacted by email up to 3 times with a personalized survey link (SurveyMonkey). We provided a $20 incentive per participant for completing the survey. We followed the American Association for Public Opinion Research (AAPOR) reporting guideline’s recommendations for Internet Surveys of Specifically Named Persons and computed the survey response rate according to standard definitions (number of surveys with complete responses divided by number of surveys [complete plus partial] plus the number of surveys with no response).^19^ Electronic informed consent was provided by participants. This study was approved by Cleveland Clinic’s institutional review board.

To assess which factors (if any) contribute to physician prioritization of preventive services, the survey asked respondents to agree or disagree (5-point Likert scale, ranging from strongly disagree to strongly agree) with the statement “I prioritize which preventive care services I will discuss with my patients.” We then presented 15 statements about prioritization and asked respondent opinions on the same Likert scale. Of these, 5 questions considered time management and limitations (eg, “In a routine office visit, I have enough time to address a patient’s preventive care needs”), 9 questions asked about specific factors (eg, “How important is each of the following when deciding which preventive care services you will discuss with your patients?”), and 1 question asked whether physicians wanted help with prioritization (“I could use some guidance on how to prioritize preventive care services.).”

To learn whether physicians vary recommendations according to visit length or patient risk factors, the survey asked physicians to envision preventive care discussions with hypothetical patients. Specifically, we developed a 2 × 2 factorial design of 2 visit lengths (40 minutes for a new patient and 20 minutes for a minor, acute illness with 5 minutes left over) and 2 hypothetical patients. Visit lengths were intended to assess how recommendations may vary across 2 common settings in which preventive care discussions occur. Each physician received all 4 scenarios. The order was randomized by visit length. That is, some physicians first received the 40-minute scenario followed by the 20-minute scenario for patient 1, then the 40-minute scenario followed by the 20-minute scenario for patient 2 (eAppendix in the Supplement). Other physicians first received the 20-minute scenario followed by the 40-minute scenario for patient 1, then the 20-minute scenario followed by the 40-minute scenario for patient 2. The scenarios, developed with clinician input, were based on common cases seen in practice. We constructed the scenarios so that US life tables, which are based solely on age, sex, and race,^20^ reported similar life expectancy for both patients. However, because these tables ignore clinical risk factors, true life expectancy differed across patients.

Hypothetical patient 1 was a 50-year-old white woman with hypertension (blood pressure of 150/90 mm Hg); with type 2 diabetes (hemoglobin A_1c_ level of 9%) and receiving metformin hydrochloride, 500 mg twice daily; with hyperlipidemia, including a total cholesterol level of 280 mg/dL, low-density lipoprotein cholesterol level of 150 mg/dL, and high-density lipoprotein cholesterol level of 40 mg/dL (to convert cholesterol to mmol/L, multiply by 0.0259); body mass index (calculated as weight in kilograms divided by height in meters squared) of 35; a 30-pack-year history of smoking to present; and a family history that included 1 first-degree relative diagnosed with breast cancer at 50 years of age. Hypothetical patient 2 was a 45-year-old black man with a generally similar medical history to patient 1 (hypertension [blood pressure, 150/90 mm Hg]; hyperlipidemia, [total cholesterol level of 280 mg/dL, low-density lipoprotein cholesterol level of 150 mg/dL, and high-density lipoprotein cholesterol level of 40 mg/dL]; body mass index of 35; and a 30-pack-year history of smoking to present); and a family history that included 1 first-degree relative diagnosed with colorectal cancer at 50 years of age. Neither patient had previous cancer screening. Both had already received a flu shot.

We reasoned that based on differences in clinical risk factors, preventive care priorities may differ across patients. For example, although both patients would benefit from cardiovascular risk reduction, physicians may question whether colorectal cancer screening should be prioritized in patient 2 because of his family history and black race. Similarly, physicians may question the relative priority of hypertension vs glycemic control in patient 1. Previous studies^14,15^ found substantial variation in the absolute benefits and harms of preventive services across hypothetical patients, affecting the rank order of services most likely to improve life expectancy.

We considered major preventive care recommendations for which each patient would be eligible per the US Preventive Services Task Force (USPSTF) (12 services for patient 1, 11 for patient 2), in alphabetical order: aspirin therapy (recommended at the time of our study)^21,22^; control of blood pressure or glucose or lipid levels; healthy diet; exercise; screening for breast cancer (patient 1 only), cervical cancer (patient 1 only), colorectal cancer, depression, lung cancer (included because of smoking history, although the USPSTF recommends screening begin at 55 years of age), or prostate cancer (patient 2 only); smoking cessation; and weight loss.^1^ Although prostate cancer screening was rated grade D (harms outweigh benefits) by the USPSTF from 2012 to 2018,^23,24^ we included it based on clinical practice. In addition, although the USPSTF and mathematical model grouped healthy diet and physical activity in a single recommendation (similar to randomized clinical trials),^25^ the survey separated these into 2 services based on clinical feedback that physicians may address them separately.

For each scenario, we presented 3 sets of questions to assess preventive care priorities. First, using a 5-point Likert scale (ranging from definitely do not discuss during this visit to definitely discuss during this visit), physicians were asked how likely they would be to discuss each preventive service. Second, physicians were asked to select the 3 most important (top 3) preventive services to address. Finally, physicians were asked to rank their top 3 priorities in order of importance. Recognizing that discussions may take place over time (eg, address some services immediately, others at follow-up visits), each question referenced the current visit.

We analyzed responses to each question asked on a Likert scale with summary statistics (means [95% CIs], number [percentage] of responses, or ≤2.00 or ≥4.00 of 5.00) and compared differences between the visit lengths and hypothetical patients with 2-tailed *t* tests. Although only extremes of the 5-point Likert scale were explicitly defined in the survey (ranging from definitely not to definitely), for analysis, we assumed 3.00 to be neutral and thus defined no more than 2.00 and at least 4.00 as unlikely and likely, respectively. After verifying that physician sex, age, years in practice, clinical full-time equivalent, and department were not significant covariates, we chose to present unadjusted results.

Finally, we compared physician recommendations with the preventive services most likely to improve life expectancy. We did this because physicians indicated that this consideration was an important determinant of their priorities and because life expectancy captures both incidence and timing of cases prevented. We used a previously published mathematical framework to individualize an expectation about the years of life gained from each preventive service recommended (grade A or B) by the USPSTF.^14,15^ For example, in the general population, breast cancer screening adds 122 life-years per 1000 individuals,^26^ or about 1.5 months per person (122/1000 life-years). To individualize the calculation, the model assigned higher benefits for women with known risk factors (eg, family history) but lower benefits for those with heavy smoking history (because of lower baseline life expectancy). In each scenario, services were rank ordered based on the potential to improve life expectancy and compared with physician selections. Analyses were conducted with STATA/MP, version 15.1 (StataCorp LLC).

## Results

Of 241 primary care physicians invited to participate, 137 (57%) responded. Median completion time was 11 (interquartile range [IQR], 8-15) minutes. Most respondents were female (74 [54%]) and younger than 50 years (85 [62%]) (Table 1), compared with 39% female and 55% younger than 55 years nationally.^27,28^ Internal medicine and family medicine were similarly represented (74 [54%] and 63 [46%], respectively), comparable to national data.^27,28^

**Table 1.** Demographic Characteristics of Participating Physicians

Characteristic	No. (%) of physicians (n = 137)^a^
Female	74 (54)
Age, y	
<40	36 (26)
40-49	49 (36)
50-64	49 (36)
≥65	3 (2)
Time in practice, y	
<5	18 (13)
5-9	22 (16)
10-20	50 (36)
>20	47 (34)
Clinical FTE, %	
<20	1 (1)
20-39	8 (6)
40-59	5 (4)
60-79	27 (20)
≥80	96 (70)
Practice type	
Internal medicine	
Academic	19 (14)
Community	55 (40)
Family medicine	63 (46)

Abbreviation: FTE, full-time equivalent.

^a^ Percentages have been rounded and may not total 100.

### Overall Use of Prioritization

Physicians strongly agreed with the statement, “I prioritize which preventive care services I will discuss with my patients.” As shown in Table 2, the mean score was 4.27 (95% CI, 4.12-4.42). Physicians generally agreed that they had enough time to address preventive care during an annual physical examination (mean score, 4.21; 95% CI, 4.02-4.40) but not a routine office visit (mean score, 2.18; 95% CI, 1.99-2.38). Similarly, 103 physicians (75%) agreed that they discussed some, but not all, preventive services recommended in the electronic health record health maintenance tab (Epic Systems Corporation) during every visit (mean score, 3.99; 95% CI, 3.78-4.19), whereas only 41 (30%) agreed that they discussed all recommended preventive services (mean score, 2.66; 95% CI, 2.44-2.89).

**Table 2.** Physicians’ Overall Views on Prioritization and Contributing Factors

Survey item	Mean score (95% CI)	No. (%) of physicians	
		Choosing ≥4.00 of 5.00 on Likert scale	Choosing ≤2.00 of 5.00 on Likert scale
**“Please tell us whether you agree or disagree with the following statements.”**^**a**^			
I prioritize which preventive care services I will discuss with my patients.	4.27 (4.12-4.42)	118 (86)	7 (5)
In an annual physical, I have enough time to address a patient’s preventive care needs.	4.21 (4.02-4.40)	112 (82)	13 (9)
In every visit, I discuss some, but not all, preventive care services in the health maintenance tab with my patients.	3.99 (3.78-4.19)	103 (75)	20 (15)
I am more likely to recommend a preventive care service if I think a patient will adhere to my recommendation.	3.07 (2.82-3.33)	65 (47)	53 (39)
I could use some guidance on how to prioritize preventive care services.	2.96 (2.75-3.18)	47 (34)	49 (36)
In every visit, I discuss all preventive care services in the health maintenance tab with my patients.	2.66 (2.44-2.89)	41 (30)	68 (50)
In a routine office visit, I have enough time to address a patient’s preventive care needs.	2.18 (1.99-2.38)	20 (15)	88 (64)
**“How important is each of the following when deciding which preventive care services you will discuss with your patients?”**^**b**^			
Ability to improve the patient’s quality of life	4.56 (4.44-4.68)	126 (92)	2 (1)
Ability to help the patient live longer	4.53 (4.40-4.66)	126 (92)	4 (3)
Strength of recommendation by professional organizations/guidelines	4.36 (4.23-4.49)	124 (91)	5 (4)
Recommended in health maintenance tab	4.17 (4.03-4.31)	113 (82)	6 (4)
Time required to discuss recommendation	3.95 (3.77-4.13)	95 (69)	14 (10)
Patient interest	3.69 (3.49-3.88)	83 (61)	21 (15)
Cost of the services	3.34 (3.13-3.56)	64 (47)	33 (24)
Probability that patient will adhere to recommendation	3.19 (2.98-3.40)	60 (44)	41 (30)

^a^ Scored from 1 for strongly disagree to 5 for strongly agree.

^b^ Scored from 1 for not at all important to 5 for very important.

### Factors Contributing to Physician Recommendations

The top-rated factors influencing physician recommendations were ability to improve patient quality of life (mean score, 4.56; 95% CI, 4.44-4.68) or length of life (mean score, 4.53; 95% CI, 4.40-4.66), followed by recommendation from the electronic health record (mean score, 4.17; 95% CI, 4.03-4.31) or professional organizations/guidelines (mean score, 4.36; 95% CI, 4.23-4.49) (Table 2). Ninety-five physicians (69%) agreed that time required to discuss the service (mean score, 3.95; 95% CI, 3.77-4.13) and 83 (61%) agreed that patient interest (mean score, 3.69; 95% CI, 3.49-3.88) were important factors. Physicians were divided on the importance of cost (mean score, 3.34; 95% CI, 3.13-3.56) and probable patient adherence (mean score, 3.19; 95% CI, 2.98-3.40), with nearly half (65 [47%] and 60 [44%], respectively) considering these factors important and 33 (24%) (for cost) to 53 (39%) (for adherence) considering them not important.

When asked whether they could use guidance on how to prioritize preventive care services, physicians were evenly divided, with 47 (34%) agreeing and 49 (36%) disagreeing (Table 2). Thirty-eight physicians (28%) provided optional free-text comments on factors influencing their preventive care recommendations. Most commonly cited factors were time constraints (n = 18) and access to health care (n = 11).

### Prioritization of Preventive Services by Physicians According to Visit Length

Table 3 and eFigure 1 in the Supplement present preventive care services endorsed by physicians in the survey. Consistent with their overall views on prioritization, physicians reported that they would prioritize only in the 20-minute visit. During the 40-minute visit, physicians stated they were likely to discuss a median of 11 (IQR, 9-13) preventive services for patient 1 and 9 (IQR, 8-11) preventive services for patient 2 (eFigure 1 in the Supplement). For both patients, every preventive service except lung cancer screening was endorsed by most physicians (Table 3). However, during a 20-minute visit, physicians stated they were likely to discuss a median of 5 (IQR, 3-8) preventive services for patient 1 and 4 (IQR, 3-6) preventive services for patient 2 (eFigure 1 in the Supplement). Only 5 preventive services for patient 1 and 4 services for patient 2 garnered endorsement by most of the physicians (Table 3).

**Table 3.** Physician Ratings of Their Likelihood of Discussing Major Preventive Recommendations During the Current Visit^a^

Recommendation	Mean score (95% CI)^b^		Physicians likely to discuss each service (≥4.00 of 5.00 on Likert scale), No. (%)^b^	
	40 min for new-patient visit	20 min for a minor, acute illness with 5 min left over	40 min for new-patient visit	20 min for a minor, acute illness with 5 min left over
Patient 1				
Glycemic control	4.95 (4.91-4.99)	4.14 (3.95-4.34)	136 (99)	100 (73)
Blood pressure control	4.94 (4.90-4.99)	4.39 (4.23-4.56)	136 (99)	118 (86)
Smoking cessation	4.87 (4.80-4.94)	4.30 (4.10-4.49)	134 (98)	109 (80)
Lipid level control	4.77 (4.67-4.87)	3.30 (3.07-3.54)	127 (93)	68 (50)
Weight loss	4.72 (4.61-4.82)	3.25 (3.01-3.48)	129 (94)	62 (45)
Breast cancer screening	4.67 (4.54-4.80)	3.31 (3.05-3.57)	128 (93)	69 (50)
Healthy diet	4.58 (4.45-4.71)	3.16 (2.90-3.42)	119 (87)	62 (45)
Exercise	4.58 (4.45-4.70)	3.04 (2.80-3.29)	120 (88)	54 (39)
Colorectal cancer screening	4.33 (4.14-4.52)	2.58 (2.32-2.84)	109 (80)	43 (31)
Cervical cancer screening	4.02 (3.80-4.24)	2.23 (1.99-2.47)	98 (72)	28 (20)
Aspirin therapy	3.88 (3.66-4.10)	2.33 (2.10-2.56)	92 (67)	28 (20)
Depression screening	3.77 (3.55-3.98)	2.33 (2.08-2.57)	80 (58)	33 (24)
Lung cancer screening	2.86 (2.60-3.12)	1.64 (1.46-1.81)	49 (36)	11 (8)
Patient 2				
Blood pressure control	4.94 (4.89-4.99)	4.61 (4.47-4.74)	136 (99)	127 (93)
Smoking cessation	4.92 (4.87-4.98)	4.64 (4.50-4.78)	136 (99)	125 (91)
Lipid level control	4.76 (4.66-4.86)	3.54 (3.32-3.76)	128 (93)	73 (53)
Colorectal cancer screening	4.73 (4.59-4.87)	3.68 (3.43-3.93)	128 (93)	84 (61)
Weight loss	4.61 (4.49-4.74)	2.93 (2.69-3.16)	128 (93)	48 (35)
Diabetes screening	4.57 (4.43-4.70)	2.77 (2.52-3.01)	122 (89)	46 (34)
Exercise	4.49 (4.34-4.64)	2.88 (2.63-3.12)	118 (86)	46 (34)
Healthy diet	4.45 (4.30-4.61)	2.93 (2.68-3.18)	115 (84)	50 (36)
Depression screening	3.56 (3.33-3.80)	2.15 (1.93-2.38)	70 (51)	24 (18)
Prostate cancer screening	3.51 (3.24-3.78)	1.99 (1.77-2.22)	77 (56)	22 (16)
Aspirin therapy	3.45 (3.18-3.71)	2.15 (1.92-2.37)	75 (55)	29 (21)
Lung cancer screening	2.92 (2.65-3.19)	1.75 (1.57-1.94)	58 (42)	12 (9)

^a^ We presented a 2 × 2 factorial of 2 visit lengths and 2 hypothetical patients who were new to the physician and were eligible for at least 11 preventive services. We then asked physicians to report their likelihood of discussing each service during the current encounter.

^b^ Scored from 1 for definitely not discuss this visit to 5 for definitely discuss this visit.

**Figure 1.**
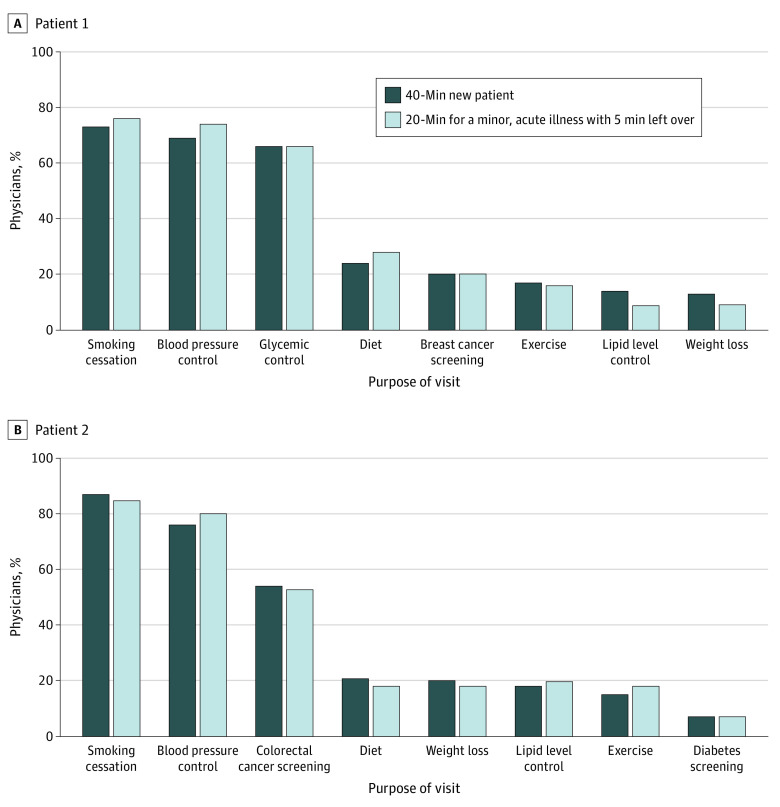
Top 3 Preventive Recommendations to Address in the Current Visit Recommendations were chosen by more than 5% of physicians.

**Figure 2.**
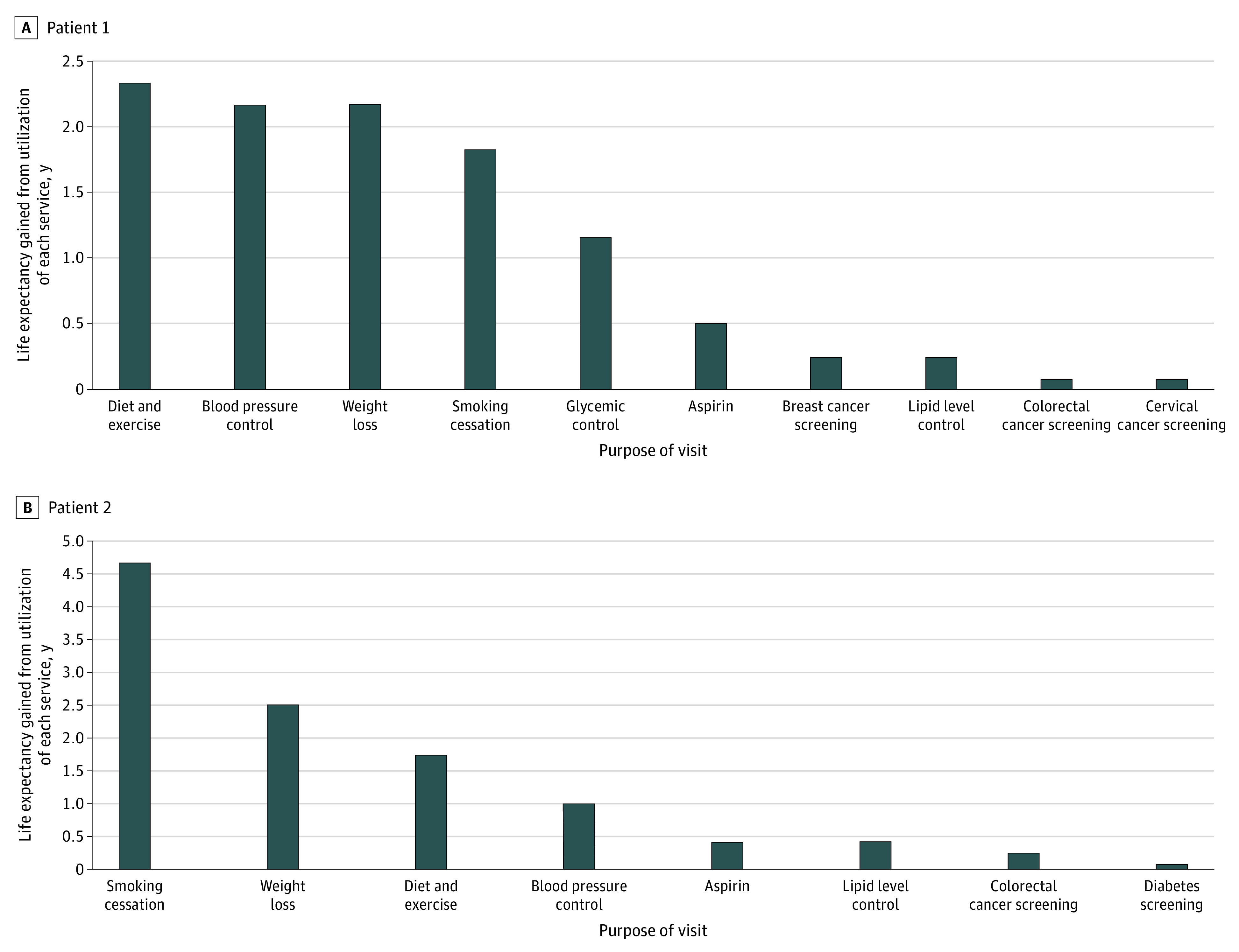
Net Expected Effect of Each Preventive Recommendation on Life Expectancy Based on Prior Literature

Because physicians reported that they only would prioritize during the 20-minute visit, we report below on 20-minute visits. Figure 1 and Figure 2 contain results for both visit lengths.

### Prioritization of Preventive Services by Physicians Across Patients

Physician recommendations were similar across patients. Among the 5 preventive services physicians were most likely to discuss during the 20-minute visit, 3 were the same across both patients: blood pressure control, smoking cessation, and lipid level control. Remaining priorities reflected differences in eligibility (glycemic control and breast cancer screening for patient 1, services for which patient 2, a man without diabetes, was ineligible) and family history (breast cancer screening for patient 1 and colorectal cancer screening for patient 2). Similarly, physicians generally agreed on their top 2 preventive care priorities, but these did not differ by patient. For patients 1 and 2, most physicians chose smoking cessation (104 [76%] and 116 [85%], respectively) and blood pressure control (101 [74%] and 110 [80%], respectively) (Figure 1). Apart from these, physicians disagreed. Only 49 (36%) chose the same top 3 priorities for patient 1 and 51 (37%) for patient 2 (eFigure 2 in the Supplement).

### Comparison of Physician Recommendations With the Preventive Services Most Likely to Improve Life Expectancy

In contrast to physician recommendations, the mathematical model determined that lifestyle changes offered strong potential to improve each patient’s longevity (Figure 2). For patient 1, 3 services would increase life expectancy by 2 years: diet and exercise, blood pressure control, and weight loss. Smoking cessation ranked fourth. For patient 2, smoking cessation offered the greatest increase in life expectancy (nearly 5 years), followed by weight loss (2.5 years) and diet and exercise. Despite his family history, colorectal cancer screening ranked seventh. Differences between patients primarily arose because of greater benefits of smoking cessation in men (2.5-fold greater benefit in 45-year-old men than in 50-year-old women).^29^ By contrast, just 48 physicians (35%) ranked a lifestyle intervention (healthy diet, exercise, or weight loss) in their top 3 recommendations for either patient, and only 15 (11%) ranked a lifestyle intervention as the top recommendation.

## Discussion

In this survey of internal and family medicine physicians at a large health care system, physicians reported prioritization of preventive services, particularly in a short visit, with key factors including potential to improve a patient’s quality or length of life. Faced with limited time, physicians stated they would recommend fewer services. Physicians’ top 3 priorities were similar across patients despite different risk factors, and on average included only 1 of the 3 preventive services most likely to improve life expectancy. Although prior work has modeled optimal prioritization of preventive services,^14,15,16,17,18^ to our knowledge, our study is the first to assess prioritization in clinical practice.

Recommendations were similar across patients, although the patients stood to benefit from different services. Although we do not know why physicians did not individualize the recommendations, they may have used rules-of-thumb, such as the importance of blood pressure control or smoking cessation, rather than attempting to identify which service would provide the greatest benefit for a specific patient. It is also likely that physicians were not aware of the fact that the same service would provide substantially different benefit to each patient. They may have had trouble estimating the benefits of specific services. This might also explain why physicians prioritized services that did not offer the greatest effect on life expectancy. In particular, physicians infrequently recommended lifestyle interventions, including exercise, diet, and weight loss, although these had the greatest potential benefit. Similarly, most recommended colorectal cancer screening for patient 2. However, despite the patient’s family history of colorectal cancer, the effect of screening on life expectancy would be less than expected, because his cardiovascular risk factors contributed to the risk of an early death. At the same time, 1 in 5 said they would discuss prostate cancer screening, which was unlikely to affect life expectancy at all. These inconsistencies suggest a need for tools to facilitate prioritization in clinical practice, although only approximately 1 of 3 physicians stated they would appreciate such help. Unfortunately, the few tools that currently exist are early stage and not ready for widespread implementation.^14,15,16,17,18,30,31^

Other factors may have influenced physicians’ choices. Nearly half of physicians reported that likelihood of patient adherence was an important factor for prioritization, which may have contributed to low prioritization of lifestyle interventions. Diet and exercise interventions are rarely successful in primary care, instead requiring intensive 9- to 12-month programs for low-to-moderate improvements,^32^ and for patients with obesity, the chance of sustaining weight loss is less than 1%.^33^ Time required to discuss lifestyle changes in the primary care setting and access to lifestyle counselors also may be barriers, reducing physicians’ prioritization. Physicians may also view lifestyle changes as contributing to blood pressure, diabetes, and lipid level control, rather than as separate goals. In addition, physicians in accountable care organizations may be incentivized to focus on attainment of quality metrics, which usually do not include diet and exercise, potentially influencing our results.^34,35,36^ In contrast, smoking cessation was a highly prioritized lifestyle change, perhaps because of a higher chance of success (3%-17% per quit attempt).^37^ We caution that while considering average adherence rates to interventions (eg, obesity control less likely than hypertension control) may be reasonable, careful consideration should be given before individualizing estimated adherence rates by patient; failure to do so could inadvertently exacerbate health disparities for minorities.

### Limitations

This study has some limitations. The response rate of 57% may result in response bias. In addition, there may have been social desirability response bias, with respondents experiencing pressure to state that they prioritized. Second, generalizability may be limited by data from a single health care system. Third, we presented hypothetical new patient visits and cannot extrapolate to follow-up visits. Fourth, the mathematical model was a previously published proof-of-concept approximation for life expectancy benefits of each preventive service and did not consider quality of life. Fifth, recent evidence suggests much lower benefits and greater harms of aspirin therapy.^38,39^

## Conclusions

In this survey study, despite consensus on overall priorities, just one-third of physicians agreed on the 3 most important services to address in a single visit. Physician recommendations did not differ substantially between patients, even when the patients faced different risk factors. Physicians did not prioritize weight loss, healthy diet, and exercise, despite large potential for gains in life expectancy. Future research may consider how health care systems can better incentivize lifestyle changes and whether physicians and patients would benefit from guidance on preventive care priorities in a time-limited setting.

## Notes

Supplement. **eAppendix.** Survey **eFigure 1.** Number of Preventive Services Physicians Were Likely to Discuss (≥4 of 5) During a 40-Minute or 20-Minute Office Visit **eFigure 2.** Most Common Combinations of the Top 3 Preventive Recommendations to Address in the Current Visit (Chosen by >5% of Physicians)
