# Supplementary material for: Assessment of Physician Priorities in Delivery of Preventive Care
Source: JAMA Netw Open. 2020 Jul 27;3(7):e2011677. doi: 10.1001/jamanetworkopen.2020.11677 (PMC8103855; doi:10.1001/jamanetworkopen.2020.11677)
Supplement: Supplement. — eAppendix. Survey eFigure 1. Number of Preventive Services Physicians Were Likely to Discuss (≥4 of 5) During a 40-Minute or 20-Minute Office Visit eFigure 2. Most Common Combinations of the Top 3 Preventive Recommendations to Address in the Current Visit (Chosen by >5% of Physicians) [file jamanetwopen-3-e2011677-s001.pdf]

# Supplementary Online Content

Zhang JJ, Rothberg MB, Misra-Hebert AD, Gupta NM, Taksler GB. Assessment of physician priorities in delivery of preventive care. *JAMA Netw Open*. 2020;3(7):e2011677. doi:10.1001/jamanetworkopen.2020.11677

**eAppendix.** Survey
**eFigure 1.** Number of Preventive Services Physicians Were Likely to Discuss (≥4 of 5) During a 40-Minute or 20-Minute Office Visit
**eFigure 2.** Most Common Combinations of the Top 3 Preventive Recommendations to Address in the Current Visit (Chosen by >5% of Physicians)

This supplementary material has been provided by the authors to give readers additional information about their work.

©2020 Zhang JJ et al. *JAMA Network Open*.

eAppendix. Survey

Survey Version A (40-minute visit then 20-minute visit for a minor, acute illness with 5 minutes left over)

# Thank you for your time.

**You are being invited to take part in a research study by the Cleveland Clinic.**

**Through Survey Monkey (internet-based software), the Cleveland Clinic is surveying eligible physicians to better understand provider attitudes toward preventive care. By taking this survey, you can help researchers understand differences in provider opinions about disease prevention.**

**What are the requirements and time commitment to participate? This study involves a survey that will take about 15 minutes.**

**What are the benefits of taking part in this study?**

**You will experience no direct benefit from participating in the research. The information from this study may improve knowledge about provider attitudes toward preventive care.**

**Are there any risks to you participating in this study?**

**This is minimal risk research. You may experience anxiety or discomfort from answering survey questions. There is minimal risk to the confidentiality of your information. Your name will NOT be linked to your responses.**

**Do I have to participate in the research?**

**Your participation is strictly voluntary. If you choose to complete the survey, you can stop at any time.**

**Will my privacy and confidentiality be protected?**

**All of your answers to this survey will be confidential. Survey Monkey will collect and store your answers, and send them to the Cleveland Clinic.**

**Will I be compensated for my time?**

**Yes, if you complete the survey, you will be provided with a$20 gift card. Per Cleveland Clinic Tax Department guidelines, this is considered taxable wages and will be reported on your pay stub. For this purpose only, your name and employee ID will be provided to the payroll department.**

**How do I indicate that I want to participate in the research? You agree to participate by completing this survey.**

**Who do I contact if I have questions about the research?**

**If you have any questions about this survey, you may contact the investigative team at (216) 444- 4590. Or, if you have questions about your rights as a research participant, call the Cleveland Clinic Institutional Review Board at (216) 444-2924.**

**Your help means a lot to us. We thank you again for taking the time to complete this survey.**

©2020 Zhang JJ et al. *JAMA Network Open*.

* Do you agree to participate in this survey?

- ◯ YES, I AGREE TO PARTICIPATE BY COMPLETING THIS SURVEY NO, I
- ◯ DO NOT AGREE TO PARTICIPATE

©2020 Zhang JJ et al. *JAMA Network Open*.

This survey consists of 2 sections. First, we will ask your views on 2 hypothetical patients. Then, we will ask you some questions about preventive care in your practice.

©2020 Zhang JJ et al. *JAMA Network Open*.

**Patient Scenarios**

**Patient #1**

**Please consider the following new patient, who presents for a 40- minute visit. She last saw a doctor 6 months ago and brings her labs from that visit.**

**50 y o white female, BMI 35, current smoker (30 pack-years), type II diabetes on metformin 500 mg BID. Not up-to-date with any cancer screenings. Recently received a flu shot at her local pharmacy.**

**BP 150/90**

**Labs (HbA1c 9%, TC 280, LDL 150, HDL 40)**

**Family history (1 first-degree relative diagnosed with breast cancer at age 50)**

©2020 Zhang JJ et al. *JAMA Network Open*.

* 1. Sometimes, because of time constraints, important services are not discussed. In your opinion, how likely would you be to discuss these services with this patient **at this visit?**

| | Definitely **not** discuss this visit | | | | Definitely discuss this visit |
|---|---|---|---|---|---|
| Eat a healthy diet | | | | | |
| Exercise regularly | | | | | |
| Glycemic control | | | | | |
| Lose weight | | | | | |
| Lower blood pressure | | | | | |
| Lower cholesterol | | | | | |
| Screen for breast cancer | | | | | |
| Screen for cervical cancer | | | | | |
| Screen for colorectal cancer | | | | | |
| Screen for depression | | | | | |
| Screen for lung cancer | | | | | |
| Take aspirin daily | | | | | |
| Quit smoking | | | | | |

©2020 Zhang JJ et al. *JAMA Network Open*.

* 2. Please consider the preventive services listed below. In your opinion, which are the 3 most important services to address **in this visit?** *(Please select 3)*

- Eat a healthy diet
- Exercise regularly
- Glycemic control
- Lose weight
- Lower blood pressure
- Lower cholesterol
- Screen for breast cancer
- Screen for cervical cancer
- Screen for depression
- Screen for colorectal cancer
- Screen for lung cancer
- Take aspirin daily
- Quit smoking

©2020 Zhang JJ et al. *JAMA Network Open*.

* 3. Please rank these services in order of importance**for this visit.**

- Eat a healthy diet
- Exercise regularly
- Glycemic control
- Lose weight
- Lower blood pressure
- Lower cholesterol
- Screen for breast cancer
- Screen for cervical cancer
- Screen for colorectal cancer
- Screen for depression
- Screen for lung cancer
- Take aspirin daily
- Quit smoking

©2020 Zhang JJ et al. *JAMA Network Open*.

## Patient #1

**Now, suppose the same new patient presents for a 20-minute visit for a minor, acute illness. You have 5 minutes left over. As a reminder, she last saw a doctor 6 months ago and brings her labs from that visit.**

**50 y o white female, BMI 35, current smoker (30 pack-years), type II diabetes on metformin 500 mg BID. Not up-to-date with any cancer screenings. Recently received a flu shot at her local pharmacy.**

**BP 150/90**

**Labs (HbA1c 9%, TC 280, LDL 150, HDL 40)**

**Family history (1 first-degree relative diagnosed with breast cancer at age 50)**

©2020 Zhang JJ et al. *JAMA Network Open*.

* 1. Still considering the same patient, how likely would you be to discuss these services**at this visit?**

| | Definitely **not** discuss this visit | | | | Definitely discuss this visit |
|---|---|---|---|---|---|
| Eat a healthy diet | | | | | |
| Exercise regularly | | | | | |
| Glycemic control | | | | | |
| Lose weight | | | | | |
| Lower blood pressure | | | | | |
| Lower cholesterol | | | | | |
| Screen for breast cancer | | | | | |
| Screen for cervical cancer | | | | | |
| Screen for colorectal cancer | | | | | |
| Screen for depression | | | | | |
| Screen for lung cancer | | | | | |
| Take aspirin daily | | | | | |
| Quit smoking | | | | | |

©2020 Zhang JJ et al. *JAMA Network Open*.

* 2. Still considering a visit with **5 minutes left over**, in your opinion, which are the 3 most important services to address **in this visit?** *(Please select 3)*

- Eat a healthy diet
- Exercise regularly
- Glycemic control
- Lose weight
- Lower blood pressure
- Lower cholesterol
- Screen for breast cancer
- Screen for cervical cancer
- Screen for depression
- Screen for colorectal cancer
- Screen for lung cancer
- Take aspirin daily
- Quit smoking

©2020 Zhang JJ et al. *JAMA Network Open*.

* 3. Still considering a visit with **5 minutes left over**, please rank these services in order of importance **for this visit.**

- [ ] Eat a healthy diet
- [ ] Exercise regularly
- [ ] Glycemic control
- [ ] Lose weight
- [ ] Lower blood pressure
- [ ] Lower cholesterol
- [ ] Screen for breast cancer
- [ ] Screen for cervical cancer
- [ ] Screen for colorectal cancer
- [ ] Screen for depression
- [ ] Screen for lung cancer
- [ ] Take aspirin daily
- [ ] Quit smoking

©2020 Zhang JJ et al. *JAMA Network Open*.

## Patient #2

**Next, please consider the following new patient, who presents for a 40-minute visit. He last saw a doctor 2 years ago and brings his labs from that visit.**

**45 y o black male, BMI 35, current smoker (30 pack-years), no significant past medical history. No current medications, never had any cancer screenings. Recently received a flu shot at his local pharmacy.**

**BP 150/90**

**Labs (TC 280, LDL 150, HDL 40)**

**Family history (1 first-degree relative diagnosed with colorectal cancer at age 50)**

©2020 Zhang JJ et al. *JAMA Network Open*.

* 1. Sometimes, because of time constraints, important services are not discussed. In your opinion, how likely would you be to discuss these services with this patient **at this visit?**

| | Definitely **not** discuss this visit | | | | Definitely discuss this visit |
|---|---|---|---|---|---|
| Eat a healthy diet | ○ | ○ | ○ | ○ | ○ |
| Exercise regularly | ○ | ○ | ○ | ○ | ○ |
| Lose weight | ○ | ○ | ○ | ○ | ○ |
| Lower blood pressure | ○ | ○ | ○ | ○ | ○ |
| Lower cholesterol | ○ | ○ | ○ | ○ | ○ |
| Screen for colorectal cancer | ○ | ○ | ○ | ○ | ○ |
| Screen for depression | ○ | ○ | ○ | ○ | ○ |
| Screen for diabetes | ○ | ○ | ○ | ○ | ○ |
| Screen for lung cancer | ○ | ○ | ○ | ○ | ○ |
| Screen for prostate cancer | ○ | ○ | ○ | ○ | ○ |
| Quit smoking | ○ | ○ | ○ | ○ | ○ |
| Take aspirin daily | ○ | ○ | ○ | ○ | ○ |

©2020 Zhang JJ et al. *JAMA Network Open*.

* 2. Still considering a **40-minute** visit, in your opinion, which are the 3 most important services to address **in this visit?** *(Please select 3)*

| | |
|---|---|
| Eat a healthy diet | ◯ |
| Exercise regularly | ◯ |
| Lose weight | ◯ |
| Lower blood pressure | ◯ |
| Lower cholesterol | ◯ |
| Screen for colorectal cancer | ◯ |
| Screen for depression | ◯ |
| Screen for diabetes | ◯ |
| Screen for lung cancer | ◯ |
| Screen for prostate cancer | ◯ |
| Take aspirin daily | ◯ |
| Quit smoking | ◯ |

©2020 Zhang JJ et al. *JAMA Network Open*.

* 3. Still considering a **40-minute** visit, please rank these services in order of importance **for this visit.**

- [ ] Eat a healthy diet
- [ ] Exercise regularly
- [ ] Lose weight
- [ ] Lower blood pressure
- [ ] Lower cholesterol
- [ ] Screen for colorectal cancer
- [ ] Screen for depression
- [ ] Screen for diabetes
- [ ] Screen for lung cancer
- [ ] Screen for prostate cancer
- [ ] Take aspirin daily
- [ ] Quit smoking

©2020 Zhang JJ et al. *JAMA Network Open*.

<u>Patient #2</u>

**Now, suppose the same new patient presents for a 20-minute visit for a minor, acute illness. You have 5 minutes left over. As a reminder, he last saw a doctor 2 years ago and brings his labs from that visit.**

**45 y o black male, BMI 35, current smoker (30 pack-years), no significant past medical history. No current medications, never had any cancer screenings. Recently received a flu shot at his local pharmacy.**

**BP 150/90**

**Labs (TC 280, LDL 150, HDL 40)**

**Family history (1 first-degree relative diagnosed with colorectal cancer at age 50)**

©2020 Zhang JJ et al. *JAMA Network Open*.

* 1. Still considering the same patient, how likely would you be to discuss these services**at this visit?**

| | Definitely **not** discuss this visit | | | | Definitely discuss this visit |
|---|---|---|---|---|---|
| Eat a healthy diet | | | | | |
| Exercise regularly | | | | | |
| Lose weight | | | | | |
| Lower blood pressure | | | | | |
| Lower cholesterol | | | | | |
| Screen for colorectal cancer | | | | | |
| Screen for depression | | | | | |
| Screen for diabetes | | | | | |
| Screen for lung cancer | | | | | |
| Screen for prostate cancer | | | | | |
| Quit smoking | | | | | |
| Take aspirin daily | | | | | |

©2020 Zhang JJ et al. *JAMA Network Open*.

* 2. Still considering a visit with **5 minutes left over**, in your opinion, which are the 3 most important services to address **in this visit?** *(Please select 3)*

- Eat a healthy diet
- Exercise regularly
- Lose weight
- Lower blood pressure
- Lower cholesterol
- Screen for colorectal cancer
- Screen for depression
- Screen for diabetes
- Screen for lung cancer
- Screen for prostate cancer
- Take aspirin daily
- Quit smoking

©2020 Zhang JJ et al. *JAMA Network Open*.

* 3. Still considering a visit with **5 minutes left over**, please rank these services in order of importance **for this visit.**

- [ ] Eat a healthy diet
- [ ] Exercise regularly
- [ ] Lose weight
- [ ] Lower blood pressure
- [ ] Lower cholesterol
- [ ] Screen for colorectal cancer
- [ ] Screen for depression
- [ ] Screen for diabetes
- [ ] Screen for lung cancer
- [ ] Screen for prostate cancer
- [ ] Take aspirin daily
- [ ] Quit smoking

# Thank you for completing this section! We are almost done.

©2020 Zhang JJ et al. *JAMA Network Open*.

## Physician Attitudes

**Next, please tell us whether you agree or disagree with the following statements.**

* In a <u>routine office visit</u>, I have enough time to address a patient's preventive care needs.

Strongly disagree ○ ○ ○ ○ ○ Strongly agree

* In an annual <u>physical</u>, I have enough time to address a patient's preventive care needs.

Strongly disagree ○ ○ ○ ○ ○ Strongly agree

* I am more likely to recommend a preventive care service if I think a patient will adhere to my recommendation.

Strongly disagree ○ ○ ○ ○ ○ Strongly agree

* In every visit, I discuss some,<u>but not all</u>, preventive care services in the health maintenance tab with my patients.

Strongly disagree ○ ○ ○ ○ ○ Strongly agree

* In every visit, I discuss <u>ALL</u> preventive care services in the health maintenance tab with my patients.

Strongly disagree ○ ○ ○ ○ ○ Strongly agree

©2020 Zhang JJ et al. *JAMA Network Open*.

# Prioritization

* Please tell us whether you agree or disagree with the following statement:

**I prioritize which preventive care services I will discuss with my patients.**

| Strongly disagree | | | | Strongly agree |
|---|---|---|---|---|
| ○ | ○ | ○ | ○ | ○ |

©2020 Zhang JJ et al. *JAMA Network Open*.

* Please tell us whether you agree or disagree with the following statement:

**I could use some guidance on how to prioritize preventive care services.**

| Strongly disagree | | | | Strongly agree |
|---|---|---|---|---|
| ○ | ○ | ○ | ○ | ○ |

©2020 Zhang JJ et al. *JAMA Network Open*.

How important is each of the following when deciding which preventive care services you will discuss with your patients?

* Patient interest

Not at all important — Very important

○ ○ ○ ○ ○

* Ability to help the patient live longer

Not at all important — Very important

○ ○ ○ ○ ○

* Strength of recommendation by professional organizations/guidelines

Not at all important — Very important

○ ○ ○ ○ ○

* Recommended in health maintenance tab

Not at all important — Very important

○ ○ ○ ○ ○

* Time required to discuss recommendation

Not at all important — Very important

○ ○ ○ ○ ○

* Probability that patient will adhere to recommendation

Not at all important — Very important

○ ○ ○ ○ ○

* Cost of the services

©2020 Zhang JJ et al. *JAMA Network Open*.

Not at all important | | | | Very important
--- | --- | --- | --- | ---
○ | ○ | ○ | ○ | ○

* Ability to improve the patient's quality of life

Not at all important | | | | Very important
--- | --- | --- | --- | ---
○ | ○ | ○ | ○ | ○

©2020 Zhang JJ et al. *JAMA Network Open*.

Other important factors - please fill in (Optional)

©2020 Zhang JJ et al. *JAMA Network Open*.

## Physician Characteristics

**Below are the final questions in the survey.**

* What is your gender?

* What is your age?

* How many years have you been in practice?

* What is your clinical FTE? Please do **not** include time spent in other (e.g., administrative) capacities.

* Do you practice in internal or family medicine?

* What is your primary work location?

In your own words, is there anything else you would like to share about how you provide preventive care for patients? (Optional)

©2020 Zhang JJ et al. *JAMA Network Open*.

**eFigure 1. Number of Preventive Services Physicians Were Likely to Discuss (≥4 of 5) During a 40-Minute or 20-Minute Office Visit**

a) Patient 1
b) Patient 2
c) Patient 1, Difference between visit lengths
d) Patient 2, Difference between visit lengths

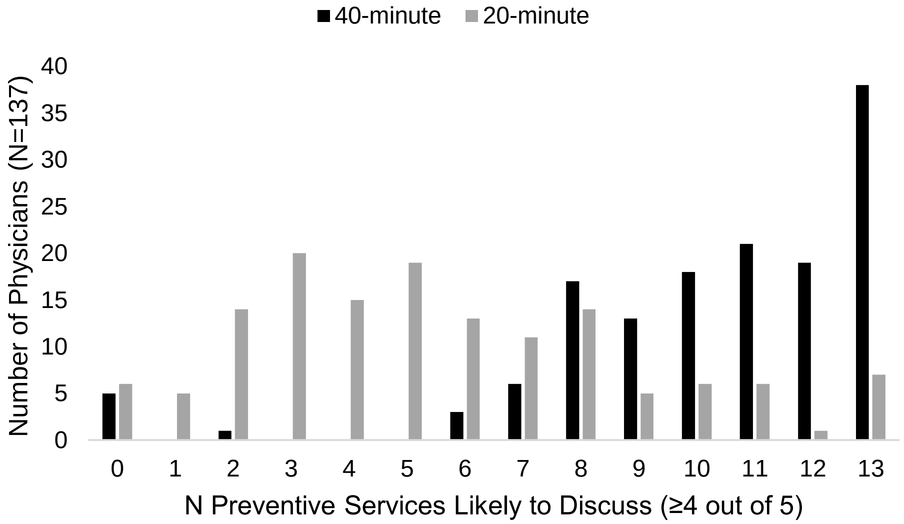


a)

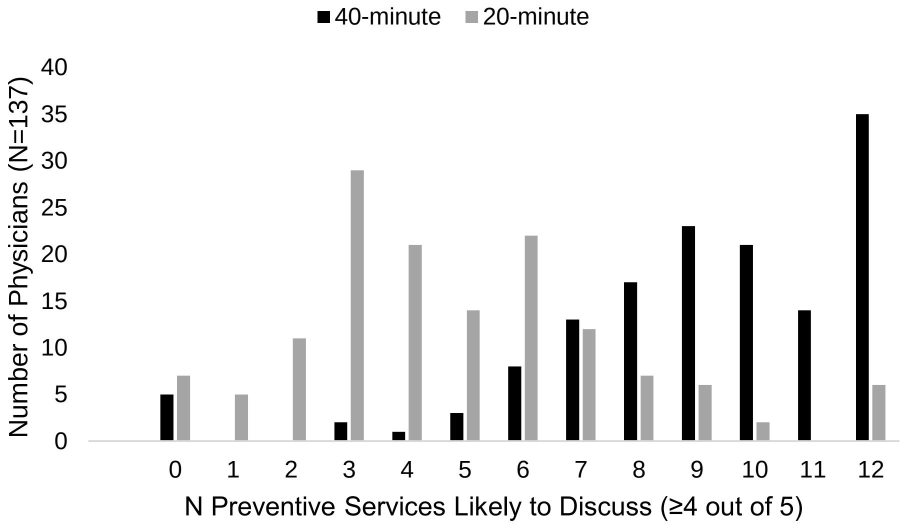


b)

©2020 Zhang JJ et al. *JAMA Network Open*.

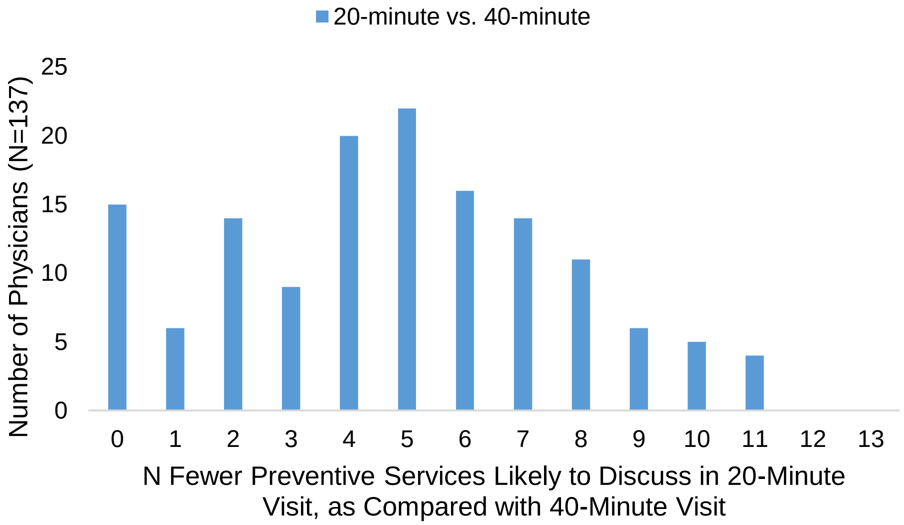


c)

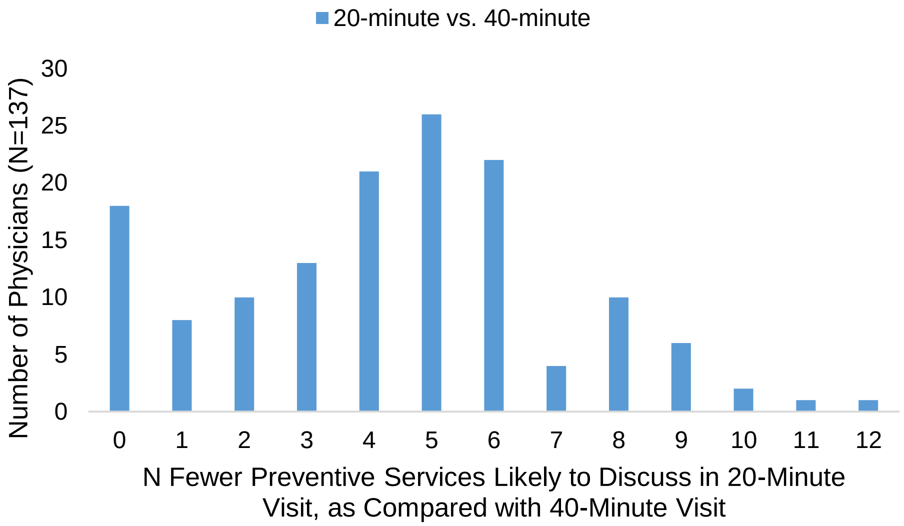


d)

©2020 Zhang JJ et al. *JAMA Network Open*.

**eFigure 2. Most Common Combinations of the Top 3 Preventive Recommendations to Address in the Current Visit (Chosen by >5% of Physicians)**

a) Patient 1
b) Patient 2

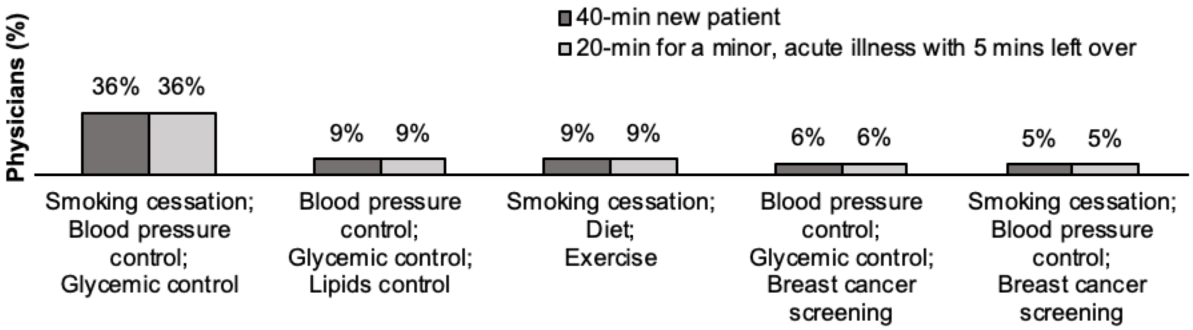


a)

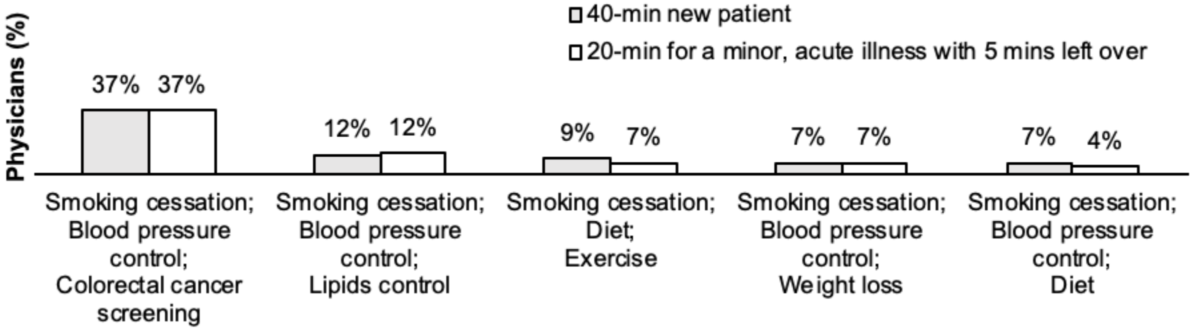


b)

©2020 Zhang JJ et al. *JAMA Network Open*.
